# Simple, Mobile-based Artificial Intelligence Algo**r**ithm in the detection of Diabetic Retinopathy (SMART) study

**DOI:** 10.1136/bmjdrc-2019-000892

**Published:** 2020-01-28

**Authors:** Bhavana Sosale, Sosale Ramachandra Aravind, Hemanth Murthy, Srikanth Narayana, Usha Sharma, Sahana G V Gowda, Muralidhar Naveenam

**Affiliations:** 1 Diabetology, Diacon Hospital, Bangalore, India; 2 Ophthalmology, Retina Institute of Karnataka, Bangalore, India; 3 Ophthalmology, Diacon Hospital, Bangalore, India

**Keywords:** retinopathy diagnosis, non-mydriatic camera, technology and diabetes, algorithms

## Abstract

**Introduction:**

The aim of this study is to evaluate the performance of the offline smart phone-based Medios artificial intelligence (AI) algorithm in the diagnosis of diabetic retinopathy (DR) using non-mydriatic (NM) retinal images.

**Methods:**

This cross-sectional study prospectively enrolled 922 individuals with diabetes mellitus. NM retinal images (disc and macula centered) from each eye were captured using the Remidio NM fundus-on-phone (FOP) camera. The images were run offline and the diagnosis of the AI was recorded (DR present or absent). The diagnosis of the AI was compared with the image diagnosis of five retina specialists (majority diagnosis considered as ground truth).

**Results:**

Analysis included images from 900 individuals (252 had DR). For any DR, the sensitivity and specificity of the AI algorithm was found to be 83.3% (95% CI 80.9% to 85.7%) and 95.5% (95% CI 94.1% to 96.8%). The sensitivity and specificity of the AI algorithm in detecting referable DR (RDR) was 93% (95% CI 91.3% to 94.7%) and 92.5% (95% CI 90.8% to 94.2%).

**Conclusion:**

The Medios AI has a high sensitivity and specificity in the detection of RDR using NM retinal images.

**Significance of this study**

**What is already known about this subject?**

- Artificial intelligence (AI) algorithms can aid in the diagnosis of diabetic retinopathy.
- These algorithms work with images taken from expensive table top fundus cameras.
- Non-availability of a fundus camera and need for high-speed internet access are limitations to their use in practice.

**What are the new findings?**

- This study evaluated the performance a new AI algorithm that works offline on a smart phone fundus camera.
- The novel ‘offline’ Medios AI algorithm had a high sensitivity for the diagnosis of referable diabetic retinopathy and sight threatening diabetic retinopathy on non-mydriatic (NM) images captured with the Remidio fundus on phone camera.

**How might these results change the focus of research or clinical practice?**

- The Medios AI and the portable Remidio NM fundus-on-phone camera together are a complete integrated solution for diabetic retinopathy detection and can make screening accessible and scalable in countries with limited resources.

## Introduction

Diabetic retinopathy (DR) is the most common cause of preventable blindness. India has close to 73 million individuals with diabetes.^1–3^ Screening and early diagnosis of DR results in early referral to the specialist, and initiation of measures to improve glycemic control and reduce progression.^4–6^

Lack of awareness, limited access to ophthalmologists, need for expensive equipment and socioeconomic barriers are challenges to screening.^1^ Although tele-ophthalmology makes screening more accessible, it is not free from challenges like the need for pupil dilatation, size and cost of fundus cameras, network connectivity issues, intergrader variability and access to ophthalmologists or trained readers.

Artificial intelligence (AI) is a potential scalable alternative in DR screening. It helps to reduce the manual burden on ophthalmologists and overcome the barriers with tele-ophthalmology. Recent advances in machine learning and convolutional neural networks has made it possible to analyze large amounts of data, recognize patterns and generate reports. AI algorithms developed for DR screening (eg, Google AI, EyeArt and IDx-DR) work on cloud-based platforms.^7–10^ The captured images are uploaded online and the algorithm provides an output within an acceptable turn over time. In low-income and middle-income countries, limited internet access or reduced bandwidth limits the use of these solutions. In addition, most cameras integrated with AI software are the traditional expensive, large fundus cameras which require the operator to capture a dilated retinal image.

The AI algorithm by Medios Technologies, Singapore is to our knowledge the first offline software for DR screening integrated with the smart phone-based fundus camera, the Remidio non-mydriatic (NM) fundus-on-phone (FOP).^11^ Studies evaluating the performance of this algorithm are limited. This study aims to evaluate the performance of an offline AI algorithm—Medios in DR screening using NM retinal images taken from the smartphone-based Remidio NM FOP retinal camera.

## Aims and objectives

### Primary aim

To evaluate the performance of the AI algorithm in detecting any grade of DR using NM retinal images captured from patients with diabetes mellitus.

### Secondary aims

To evaluate the performance of the AI algorithm in detecting referable diabetic retinopathy (RDR). RDR is defined as presence of disease greater than moderate non-proliferative DR or the presence of diabetic macular edema (DME). In addition, the ability of the algorithm to correctly identify all cases identified as STDR (severe NPDR or more severe disease or the presence of DME) by image diagnosis was also evaluated.

## Methods

The study was carried out as per the tenets of the Declaration of Helsinki (NCT03572699). Informed consent was provided was all participants enrolled in the study.

This study prospectively enrolled patients attending the outpatient department of Diacon Hospital, a university recognized, tertiary center for diabetes care and research, Bangalore, India between July and November 2018. All subjects, above the age of 18 years, with diabetes mellitus were invited to enroll for the study. Eyes with significant media opacity such as corneal opacity or cataract that precluded retinal imaging were excluded and those with known retinal vascular (artery or vein) occlusion were excluded. Enrollment continued until gradable retinal images were obtained from 900 patients. All consenting individuals meeting the inclusion criteria were screened for DR as part of routine care.

### Retinal image acquisition

Undilated retinal images were captured using the smartphone based ‘Remidio FOP camera’ (Remidio Innovative Solutions, Bangalore, India) by a trained technician. Two images (ie, disc centered (nasal field) and macula centered (posterior pole)) were captured from each eye of each patient. The technician was trained to recognize the characteristics of an excellent image and was urged to capture more than one image per field of view if required to obtain excellent images. Two additional attempts were allowed to capture the image if the image was of poor quality (eg, an out-of-focus image, or in those with a small pupil).

### Image grading by retina specialist

The de-identified (ie, anonymized) images with the subject ID were uploaded online from the FOP to an Amazon Web Services (AWS) hosted cloud service provided by the manufacturer. The images were accessed from the cloud by five retina specialists, that is, three fellowship-trained vitreoretinal surgeons and two medical retina specialists. The retina specialists individually graded the set of four retinal photographs from every eye using the International Clinical Diabetic Retinopathy Classification Severity Score.^12^ Images were graded as no DR, mild non-proliferative DR (mild NPDR), moderate non-proliferate DR (moderate NPDR), severe non-proliferate DR (severe NPDR) and proliferate DR (PDR). The images with DR were then evaluated for DME. The diagnosis of diabetic macular edema (DME) was graded as no DME, mild DME, moderate DME and severe DME. The eye with the more severe stage of retinopathy was considered as the final diagnosis for that patient, in cases where each eye had a different stage of disease severity. Patients whose images were considered as ungradable by the retina specialists were excluded from the final analysis. The majority diagnosis of the five graders was considered as the final image diagnosis. The patient-wise diagnosis obtained from the retina specialists were considered as gold standard for comparison. Each retina specialist was blinded to the diagnosis of the others and to the diagnosis of the AI.

### Image analysis using AI-based offline software

The images captured from the subjects were run offline on the iPhone6 using the Medios AI and the diagnosis was recorded in binary as DR present or absent.

#### Description of the AI software

The AI diagnosis system developed by Medios Technologies is based on Convolutional Neural Networks. It consists of a first neural network for image quality assessment and two other distinct neural networks that detect DR lesions. A final per-patient DR diagnosis is computed from the outputs of both DR neural networks and applied on all images of that patient.

Image processing is applied before feeding the images to the neural networks. The images are cropped by removing the black border surrounding the circular field of view typical of retinal images. They are resized to a common 512×512 pixels resolution. The neural network responsible for quality assessment is based on a MobileNet architecture. It consists of a binary classifier trained with images deemed as ungradable as well as with images deemed of sufficient quality. If the output is negative, a message prompts the user to recapture the image.

The other two neural networks are based on an Inception-V3 architecture and have been trained to separate healthy images from images with referable DR (moderate NPDR and above). The final output is a binary recommendation of referral to an ophthalmologist. No mild NPDR images have been used during training of the AI. The system has thus been engineered to maximize the sensitivity for referable DR and the specificity for any DR. Both networks independently analyze the images. One uses images that have been preprocessed by a contrast enhancement image processing algorithm, while the other does not. A linear classifier merges outputs of both networks into a final per-image prediction. A patient is deemed as a referable case if the prediction for one or more images is positive.

A comprehensive dataset consisting of images taken in a variety of conditions has been used for training, with a proportion of it taken using NM and/or low-cost cameras. These include 4350 NM images taken during screening camps with the Remidio FOP, and 14 266 images captured with a KOWA vx-10 mydriatic camera and 34 278 images come from the EyePACS dataset. Half of the training set contained DR cases, and the other half healthy ones.

Neural networks traditionally run on computationally powerful servers to which the end user connects and sends images. In this case, the neural network is deployed directly on the phone, leveraging smartphone technologies to make full usage of the inbuilt hardware. The whole AI diagnosis pipeline runs offline on the iPhone of the Remidio NM FOP. ‘Offline’ refers to the computational unit on which AI inference is performed. Thanks to leveraging on the high-performance capabilities of the smartphone with Core Machine Learning platforms and Open Graphics Library, image processing is done directly on the Graphics Processing Unit instead of relying on a connection to a server on the internet. There is no degradation in performance of the algorithm as a result of deploying it offline versus online. This is because, the offline mode is primarily a method of deployment that uses the smartphone to run the same algorithm as it would have on a cloud server. With newer updates, continuously trained models can be deployed through the app store, which will enable the model to get the best inferencing convenience, re-training and continuous deployment using the iPhone as a platform.

The interface and the report also provide a visual representation of the areas of the retinal images that are responsible for a positive diagnosis. This is based on a deep learning technique called class activation mapping.

Two distinct datasets have been used for internal validation and fine-tuning of the linear classifier. Both datasets had not been used for training and consist of images taken in the mydriatic mode of the camera. One dataset was captured at Dr Mohan’s Diabetes Specialities Center in Chennai, while the other was captured at Diacon Hospital in Bangalore. These results were computed independently of the institutions who provided the data. The datasets consisted of 3038 and 1054 images, respectively. The images used for training and internal validation of the AI do not overlap with those captured for the SMART study.

### Outcome measures

The primary aim was to determine the sensitivity, specificity, positive predictive value (PPV) and negative predictive value (NPV) of the AI algorithm in detecting all DR compared with the gold standard diagnosis by retina specialists. The secondary aims were to determine the sensitivity, specificity, PPV and NPV of the algorithm in the diagnosis of RDR. RDR was defined as moderate NPDR or more severe disease or the presence of DME. The ability of the algorithm to correctly identify all cases identified as sight threatening DR (STDR) by image diagnosis was also evaluated. STDR was defined as severe NPDR or more severe disease or the presence of DME.

### Statistical analysis

The Food and Drug Administration (FDA) mandated superiority cut-offs (for AI algorithms for DR screening) for sensitivity and specificity were 85% and 82.5%.^7^ The sample size required for a sensitivity of 85%, given a sensitivity of 75% under the null hypothesis using a one-sided test, 0.025 alpha and 90% power was 171 individuals with RDR. The sample size required for a specificity of 82.5% given a specificity of 75% under the null hypothesis using a one-sided test, 0.025 alpha and 90% power was 682 individuals with no RDR (combined no DR and mild NPDR). The minimum sample required was 853 and we planned to continue enrollment until gradable images could be obtained from 900 individuals.

All data were stored in Microsoft Excel and was analyzed using StataCorp V.14.2. The diagnosis of the AI was tabulated against the image diagnosis (reference standard) by constructing 2×2 tables. The sensitivity, specificity, PPV and NPV with 95% CIs were calculated, and area under the curve (AUC) plotted for all DR and RDR. In individuals diagnosed with STDR, the sensitivity (ability of the AI to correctly identify those with disease) was measured. Intergrader agreement was measured by calculating the kappa statistic.

## Results

**Figure 1:**
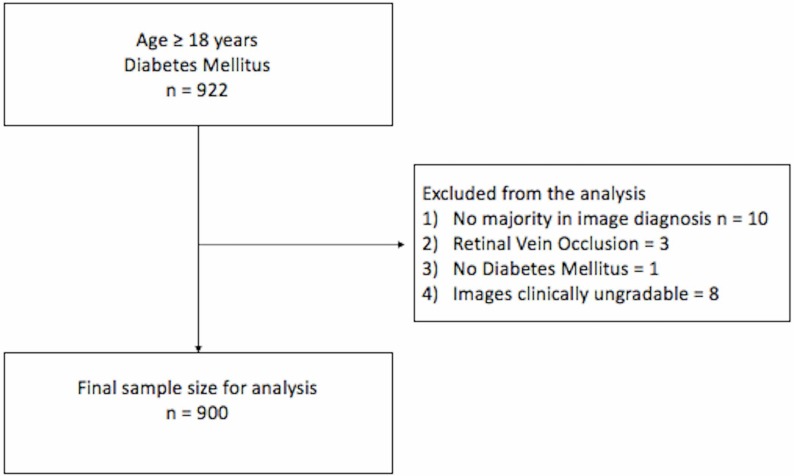
Flow chart depicting study enrollment.

The study enrolled 922 patients and the analysis included images from 900 patients (figure 1). Based on the image diagnosis, there was no evidence of DR in 648 participants (72%). Mild NPDR was seen in 51 (5.67%), moderate NPDR in 163 (18.11%), severe NPDR in 3 (0.33%) and PDR in 35 (3.89%). Mild DME was present in 12 (4.76%), moderate DME in 32 (12.69%) and severe DME in 3 (1.19%) individuals with DR with different grades of non-proliferative or proliferative DR. The intergrader agreement (quadratic weighted kappa) between the individual ophthalmologists and the majority diagnosis was between 0.79 and 0.91. Common causes of differences in diagnosis between retina specialists and the majority diagnosis were missed single microaneurysms (MA), and differentiating dot hemorrhages from MA.

**Figure 2:**
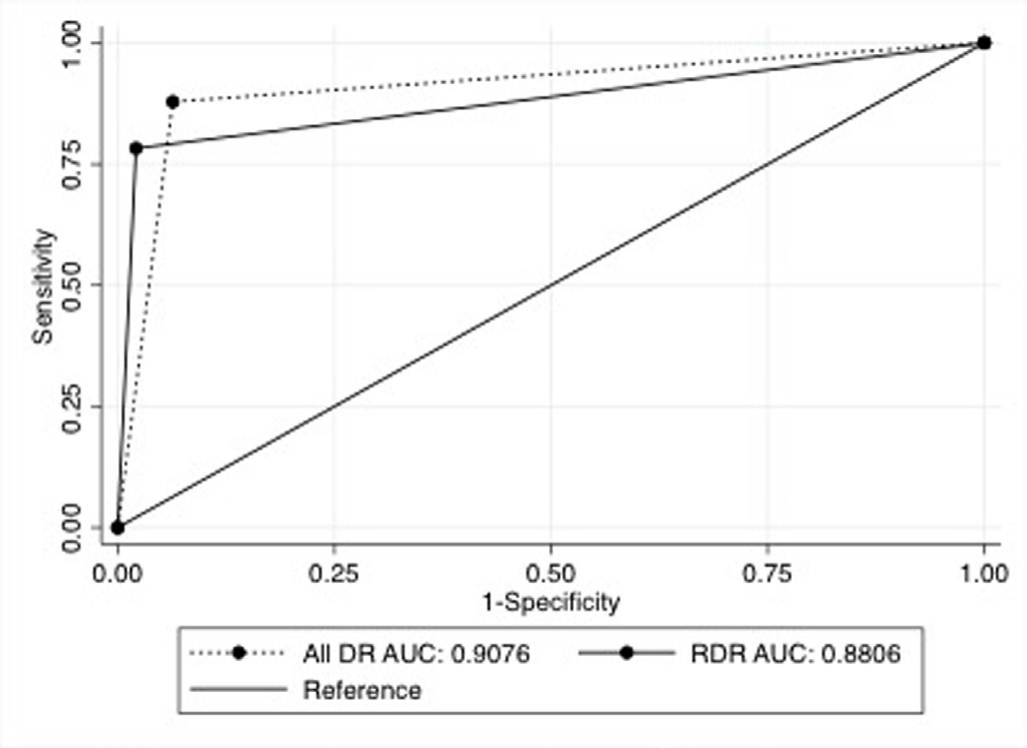
Area under the curve (AUC) of the Medios artificial intelligence algorithm for all diabetic retinopathy (all DR) and referable diabetic retinopathy (RDR).

**Figure 3:**
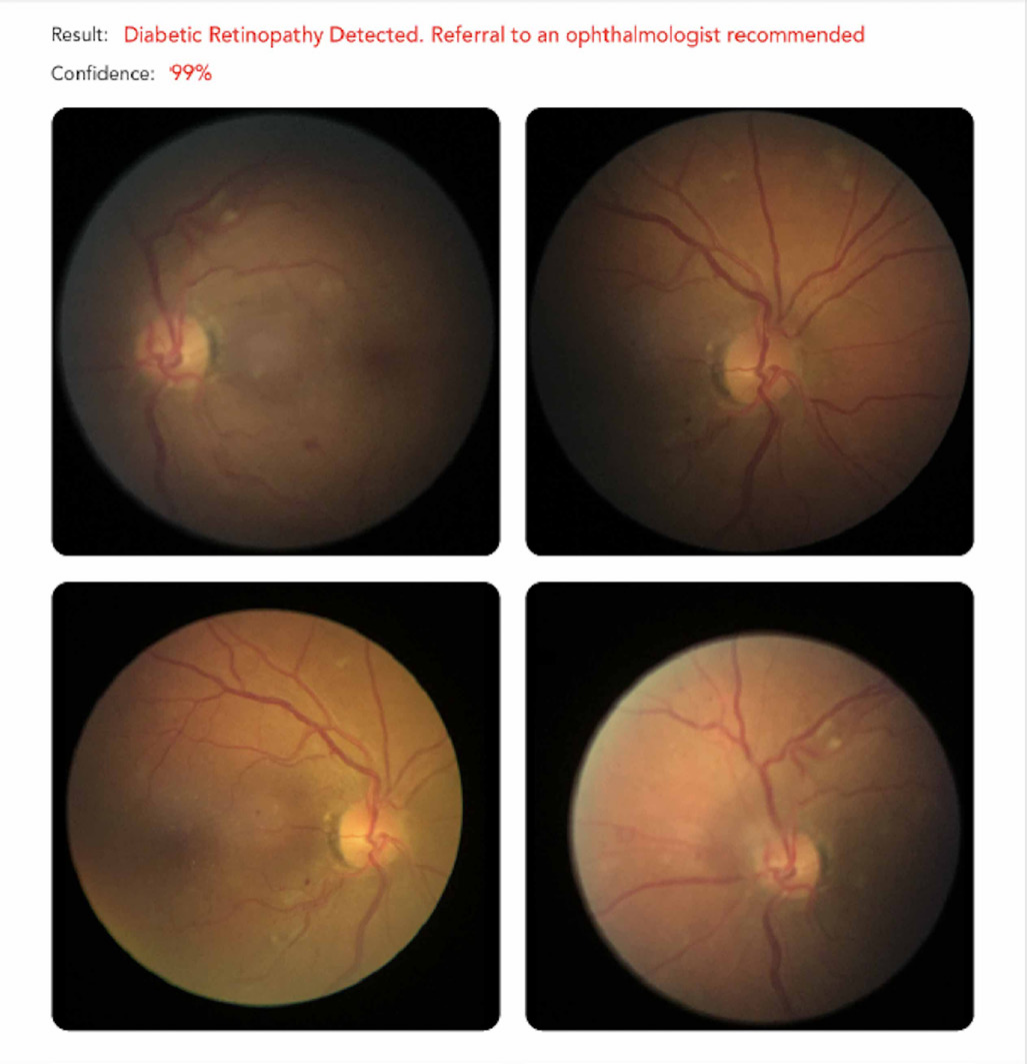
Example of the output of the Medios artificial intelligence algorithm in an individual with a diagnosis of referable diabetic retinopathy.

The AI classified 239 (26.5%) of images as DR and 661 (73.4) % as no DR. The performance of the AI in detecting all DR and RDR is summarized in table 1. The AUC for all DR and RDR are shown in figure 2. An example of the output from the Medios AI algorithm with an image diagnosis of RDR is shown in figure 3.

**Table 1:** Performance of the Medios AI

	Sensitivity (95% CI)	Specificity (95% CI)	PPV (95% CI)	NPV (95% CI)	AUC
All DR	83.3% (80.9% to 85.7%)	95.5% (94.1% to 96.8%)	87.8% (85.7% to 90%)	93.6% (92% to 95.2%)	0.9
RDR	93% (91.3% to 94.7%)	92.5% (90.8% to 94.2%)	78.2% (75.5% to 80.9%)	97.8% (96.9% to 98.8%)	0.88

AI, artificial intelligence; AUC, area under the curve; DR, diabetic retinopathy; NPV, negative predictive value; PPV, positive predictive value; RDR, referable diabetic retinopathy.

The AI was able to correctly diagnose 76/80 cases graded as STDR as having signs of retinopathy. Sensitivity for STDR was 95.2% (95% CI 88.2% to 98.6%). The three PDRs missed by the AI were postlaser images with no active changes visible. When these three images were excluded, the AI correctly identified 76/77 cases of STDR as having signs of retinopathy. The sensitivity for STDR was found to be 98.7% (95% CI 92.9% to 99.7%).

The kappa that is, agreement between the AI and the opthalmologists’ diagnosis was found to be 0.8.

## Discussion

To our knowledge, this is the first study to evaluate the performance of an AI algorithm for DR screening using NM images captured from a portable smartphone-based fundus camera. The analysis from this large study showed that the Medios AI has a high sensitivity in the detection of RDR and STDR.

The use of AI algorithms as fully automated screening solutions for DR diagnosis is on the rise. The only one to have made it past FDA’s cut is IDx-DR on the basis of a clinical study conducted with mydriatic retinal images obtained from 900 individuals. In this study, the sensitivity and specificity of the IDx-DR system in identifying RDR was 87% and 90%, meeting the FDA superiority sensitivity and specificity cut-offs of 85% and 82.5%, respectively. Despite its accuracy, it is not recommended for evaluating rapidly progressive DR.^7^ Limitations include the need for integration with expensive traditional fundus cameras. The Iowa Detection Program, a clinical study conducted to evaluate IDx-DR V.X2.1, from mydriatic retinal photographs, showed a sensitivity of 96.8% and specificity of 87.0%.^8^

The performance of the Google AI was studied on the EyePACS-1 and Messidor 2 datasets. In the EYE-PACS dataset, the sensitivity and specificity of the algorithm for RDR was 90.1% (95% CI 87.2% to 92.6%) and 98.2% (95% CI 97.8% to 98.5%). In the Messidor 2 dataset, the sensitivity and specificity was 86.6% (95% CI 80.5% to 90.7%) and 98.4% (95% CI 97.5% to 99%) for the detection of RDR.^10^ In a prospective study, the Google AI was validated across two sites in India. The sensitivity and specificity of the algorithm for the detection of RDR at Aravind Eye Hospital was 88.9% (95% CI 85.8% to 91.5%) and 92.2% (95% CI 90.3% to 93.8%); and 92.1% (95% CI 90.1% to 93.8%) and 95.2% (95% CI 94.2% to 96.1%) at Shankara Nethralaya.^13^

EyeArt (Eyenuk, Woodland Hills, California, USA) using dilated retinal images of 296 patients captured by the Remidio NMFOP was validated by Rajalaksmi *et al*. The authors reported a sensitivity of 95.8% and specificity of 80.2% for detecting any DR and a sensitivity of 99.1% and a specificity of 80.4% for detecting STDR.^14^ In a recent retrospective study, Bhaskaranand *et al* reported a sensitivity and specificity of 91% using EyeArt on 101 710 individuals.^9^ Another study by Ting *et al* with multiple retinal images taken with conventional fundus cameras from multiethnic cohorts of people with diabetes, reported a sensitivity and specificity for identifying RDR of 90.5% and 91.6%.^15^

Most of these AI algorithms require high-speed computational power and internet access for immediate reporting, in addition to the need for expensive desktop fundus cameras. This sets the Medios AI apart from other AI solutions, in being the first offline end-to-end solution integrated on the smart phone camera.

The Remidio FOP is an FDA510k cleared medical device validated in head-to-head studies against Topcon TRC 50DX and Zeiss FF450. It is the only smartphone-based device shown to have a high sensitivity and specificity in detection of all grades of DR, in non-mydriatic imaging.^11 16^ The results seen with the Medios AI using images captured from the Remidio FOP meet the FDA superiority cut-offs and are comparable to results observed with other AI algorithms, such as Google AI, EyeArt or IDx-DR for the detection of RDR.^7 9 13^ In a previous study by Rajalakshmi *et al* published in *Eye*, the Eyenuk AI algorithm, EyeArt was found to have very high sensitivity and specificity for detection of RDR and STDR when used on the Remidio FOP camera images (despite EyeArt not having been earlier trained on the Remidio FOP images).^14^

A recent study by Natarajan *et al* evaluated the performance of the Medios AI using dilated retinal images captured using the Remidio FOP from 231 individuals with diabetes. The images were captured by a healthcare worker in a primary healthcare community screening camp. The authors reported that the sensitivity and specificity of the AI in the diagnosis of RDR as 100% and 88.4%, and for any DR as 85.2% and 92%.^17^

There are a few differences between the study done by Natarajan *et al* and our study.^17^ Natarajan *et al* evaluated the AI’s performance using mydriatic images taken during community screening by healthcare workers. A sensitivity analysis was performed to assess the AI’s performance using both good quality images, and images that did not meet the minimum quality standards of the AI. In this analysis, the sensitivity of the AI for RDR remained unchanged, while the specificity dropped to 81.9%. The increase in false positive outputs were attributed to image quality. This did not translate to a concern regarding patient safety as all individuals with RDR were detected by the AI. In contrast, our study used NM images captured in a clinic setting by a trained camera technician on a larger number of individuals. Both studies demonstrate a high sensitivity and specificity for the detection of RDR. The ease of use of the device by a community healthcare worker, and results observed with both mydriatic and NM images support the use of smart phone fundus imaging and AI-based reporting for DR screening.

Cloud-based AI algorithms require internet access for real time reporting. In countries like India, where mass screening is the need of the hour, access to continuous electricity and internet is a constraint. The FOP with inbuilt offline AI can address these operational challenges in rural and urban areas in the low-income and middle-income countries with limited resources. The offline mode of AI is advantageous in the context of clinical work flow and ground deployment constraints, to ensure that DR screening can move forward without interruptions.

In this study, we observed that the AI was unable to identify laser marks as ‘DR’ in those who had ‘no active DR changes’ post pan-retinal photocoagulation. It is worthwhile to note that other studies exclude individuals who have undergone laser treatment and hence it is not possible to ascertain if other AI algorithms also behave similarly.^7 13 18 19^ Considering the practical application of these AI algorithms to be in primary care and screening (and not in tertiary hospitals visited by those with DR postlaser treatment), this finding in no way should undermine the robustness of this algorithm’s performance.

Images from eight individuals were considered clinically ungradable (figure 1). The AI algorithm had flagged six of these images as poor quality, but did provide an output for these images. Since there was no ground truth for comparison with, these images were excluded from the analysis. However, the ability to identify signs of disease pathology invisible to the human eye in less than ideal conditions—a trait of deep learning algorithms—deserves merit. In order to report if AI algorithms perform better than a clinician on poor quality/hard to grade images, it may be necessary to analyze this with a larger pool of ungradable images. Hence, at this point in time, in a real-life situation, during screening, it is necessary to use caution, and refer cases where the AI quality check flags the image as poor quality.

Limitation of our study was that it only included NM images. Hence, screening in elderly or in those with a small pupil (<3 mm) can be a challenge. Dilatation with a drop of 1% tropicamide solution may be necessary in these cases. The strengths of the study include prospective validation of the AI in a large sample against the diagnosis of five retinal specialists. Future studies that assess the performance of the AI compared with the adjudicated diagnosis, the clinical diagnosis and studies that evaluate integration of the AI into the clinical workflow are needed. We acknowledge that the AI in its current version works only integrated with the FOP and has the ability to only provide a diagnosis of referral versus no referral. Even though the algorithm is currently unable to give an output of STDR directly, we believe that every end-user should be aware of the ability of the AI to correctly identify those with STDR (at the highest risk of vision loss) and be aware of the rates and reasons of a missed diagnosis in its current version. The AI is currently being trained to grade DR, provide a diagnosis of DME and STDR in the future versions (which will continue to be deployed offline on the iPhone), to assist in triaging and immediate referral.

Our study is the first in validating the use of Medios AI in a large clinical setting using NM images. Our results show that the AI has a high sensitivity and specificity in the detection of RDR. This is the only AI system that works offline and produces real time reports on a smart phone. Multiple large-scale studies that validate the algorithm are necessary. If results are reproducible in both the mydriatic and NM setting, the Medios AI has the potential to be the scalable solution to make DR screening accessible at the primary care level.
